# Effect of virtual reality distraction on children’s behavior during dental treatment: a systematic review of the literature

**DOI:** 10.1186/s12903-026-08646-3

**Published:** 2026-05-26

**Authors:** Imane Hamdane, Maria Mtalsi, Fatima Azzahra El Mouatarif

**Affiliations:** 1 Department of Surgical Odontology, Mohammed VI Faculty of Dental Medicine, Mohammed VI University of Health Sciences, Mohammed VI Foundation for Health Sciences, Casablanca, Morocco; 2 Department of Pediatric Dentistry, Faculty of Dental Medicine, Hassan II University, Casablanca, Morocco; 3 Department of Pediatric Dentistry, Mohammed VI Faculty of Dental Medicine, Mohammed VI University of Health Sciences, Mohammed VI Foundation for Health Sciences, Casablanca, Morocco

**Keywords:** Virtual reality, Dental anxiety, Behavior, Child, Dental treatment

## Abstract

**Background:**

Dental anxiety in children is common and often disrupts dental treatment. Among various psycho-behavioral strategies, distraction techniques—especially virtual reality (VR) have gained increasing attention due to their immersive potential in managing anxiety during dental procedures.

**Methods:**

A literature search was conducted across five databases (PubMed, ScienceDirect, Google Scholar, Scopus, and Cochrane Library) for studies published between 2013 and 2023. Only randomized controlled trials and interventional studies assessing VR distraction in pediatric dental settings were included.

**Results:**

Out of 236 identified articles, 15 met the inclusion criteria, encompassing 1,168 children aged 4 to 15. Most studies reported improved behavior, reduced anxiety, and lower pain perception with VR distraction compared to standard care.

**Conclusion:**

VR distraction appears to be a safe and non-invasive approach that may improve the dental experience for children. However, due to methodological heterogeneity and the overall low certainty of evidence, these findings should be interpreted with caution, and further standardized research is needed to confirm its benefits and optimize clinical protocols.

**Systematic review registration number:**

This systematic review was based on the methodology of the Cochrane review and followed the PRISMA (preferred reporting items for systematic reviews and metanalyses) guidelines. Before starting this work, the systematic review was declared on the PROSPERO international register of systematic reviews (CRD42023458556) (https://www.crd.york.ac.uk/prospero/) to avoid duplication of subject matter by another author.

**Clinical trial number:**

Not applicable.

**Supplementary Information:**

The online version contains supplementary material available at 10.1186/s12903-026-08646-3.

## Introduction

Dental fear and anxiety in children are defined as the fear and anxiety associated with dental care. It exists in a considerable proportion of children and adolescents and constitutes a major dilemma in the practice of pediatric dentistry [1]. It is generally the result of a traumatic experience experienced by the child themselves: the child may have been subjected to an unpleasant experience due to a lack of explanation, overly blunt and non-empathetic behavior on the part of the practitioner, or a failure to optimize pain management in the dental chair.

In terms of prevalence, anxiety is ubiquitous internationally. According to a study conducted by Elmouatarif F et al. [2], 5.3% (DAS 13 to 17) of children aged 8–13 felt moderately anxious, leading 1.7% of them to refuse any clinical examination altogether, and 24% of children aged 4–8 were found to be anxious.To manage these problems of anxiety and pain, traditional methods involve the use of verbal communication, such as storytelling, audio or TV programs, or toys. As these techniques are very practitioner- and patient-dependent, their effectiveness on anxiety and pain is nevertheless rather limited. One of the best-known non-pharmaceutical approaches is distraction, whose success in medical settings is well documented. One of today’s most popular distraction techniques, with which children will be familiar and receptive, is based on computer technology that simulates the physical presence of an individual in a computer-generated 3D environment: virtual reality distraction. The child is immersed in a 3D environment through the use of a headset or 3D glasses, allowing the user to integrate a game program or a walk in a virtual world. Although some studies argue in favor of the use of virtual reality distraction methods in the management of children, as is the case in the clinical trial by Longkuan Ran et al. [3], others remain negative as to their efficacy: the clinical trial by Almajed OS et al. [4] judges the results to be of very low level of evidence and does not support the clinical interest of these virtual reality distraction techniques in the management of acute pain in children. Given the variability of the evidence in this regard, and in order to better identify the role of virtual reality distraction in managing child behavior in the dental office, a systematic review of the literature is carried out in order to answer the following questions:

Does distraction-based management using virtual reality improve children’s behavior during dental care? How effective is it compared with conventional psycho-behavioral approaches and other distraction techniques?

## Materials and methods

This systematic review was based on the methodology of the Cochrane review and followed the PRISMA (preferred reporting items for systematic reviews and metanalyses) guidelines. Before starting this work, the systematic review was declared on the PROSPERO international register of systematic reviews (https://www.crd.york.ac.uk/prospero/) to avoid duplication of subject matter by another author.

The review was conducted in accordance with the registered PROSPERO protocol and no deviations from the predefined objectives, eligibility criteria, or outcomes were made during the conduct of the review.

### Search strategy

All studies meeting the following criteria were included: studies published between 2013 and 2023, randomized controlled trials, cohort studies, and interventional studies evaluating the effects of virtual reality distraction on children’s behavior, fear, and anxiety levels during dental care.

Excluded from this review were articles written in languages other than English, meta-analyses and systematic reviews, studies including patients with systemic or syndromic conditions (“general pathologies” was replaced for clarity), and clinical studies evaluating virtual reality in medical disciplines other than pediatric dentistry.

Five electronic databases were searched: PubMed, ScienceDirect, Scopus, Cochrane Library, and Google Scholar. Google Scholar was used as a supplementary source to identify additional relevant studies due to its broader coverage, although its limitations in reproducibility and indexing are acknowledged.

The study design followed the PICO framework (Patient, Intervention, Comparison, Outcome) (Table 1).

**Table 1:** Description of PICO criteria

Population	Children (under 15 years of age) undergoing dental treatment.
Intervention	Use of virtual reality as a distraction technique during dental treatment
Comparison	Conventional psycho-behavioral management Techniques, non-VR distraction techniques or no distraction.
Results	Pain, anxiety, behavior, physiological parameters

### Search terms

To identify eligible articles, we organized the search terms into blocks corresponding the objective of this study (Table 2).

**Table 2:** Search strategy

Block 1: Distraction techniques
AND Block 2: (Virtual reality OR Virtual reality exposure therapy)
AND Block 3: (behavior control OR behavior therapy)
AND Block 4: (Dental anxiety OR dental phobia OR dental fear)
AND (Block 5: (Child OR pediatric OR paediatric OR children)
AND Block 6: (Dental care for children OR Pediatric dentistry OR Dental treatments OR Pedodontics)

All the articles included in this systematic review investigated the effects of virtual reality distraction on behavior, fear, anxiety, and pain levels in children during dental care.

The search strategy primarily relied on free-text keywords combined using Boolean operators (AND, OR). When available, controlled vocabulary terms (e.g., MeSH terms) were also incorporated.

To improve transparency and reproducibility, the complete database-specific search strategies, including Boolean operators, keyword combinations, and database-specific adaptations, are provided in Supplementary Table S1. The full PubMed search strategy, including MeSH terms, filters, and the date of the final search, is also detailed in this supplementary material.The search strategies were designed to ensure transparency and reproducibility and were adapted to the specific requirements of each database, including the use of controlled vocabulary when applicable.

The full database-specific search strategies, including Boolean operators, MeSH terms, and filters, are provided in Supplementary Table S1 to ensure reproducibility.

### Data extraction

Two independent reviewers performed data extraction to ensure accuracy and minimize bias. Extracted data included participant characteristics (age, health status), intervention details (type of virtual reality system, session duration and number), outcome measures (pain, anxiety, behavioral responses), and study results.

Discrepancies between reviewers were resolved through discussion, and when consensus could not be reached, a third senior reviewer was consulted.

A standardized data extraction form was used to ensure consistency across studies.

## Results

### Study selection

**Fig. 1:**
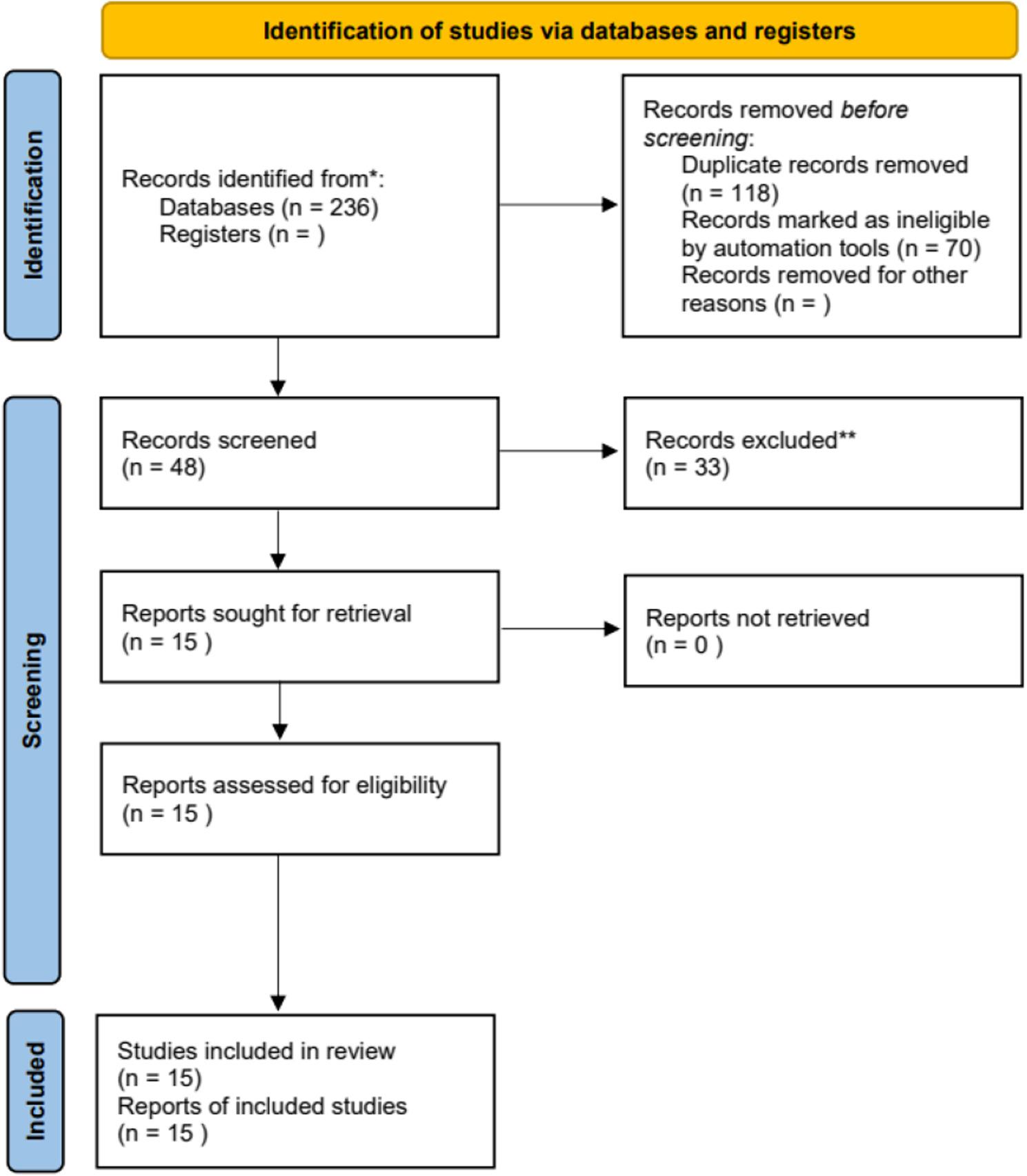
PRISMA flow chart

The database search initially identified 236 records. Duplicate records (*n* = 118) were identified and removed using the reference management software Zotero. An additional automated filtering step was applied prior to screening to remove ineligible records (*n* = 70) based on predefined criteria. Also, after deduplication and preliminary filtering, 48 records remained for title and abstract screening. Of these, 33 records were excluded, and 15 reports were sought for retrieval. All reports were successfully retrieved and assessed for full-text eligibility. Cohort studies were considered eligible during the screening process; however, no cohort studies met the inclusion criteria and none were included in the final analysis. Following full-text assessment, 15 studies met the eligibility criteria and were included in the final review. The study selection process is illustrated in Fig. 1.

### Characteristics of included studies

The 15 articles selected are published in English and are listed in a summary table specifying the country of origin, date of publication, title, type of study and keywords. We remind readers that the numbers assigned to the articles in (Table 3) are used for the presentation of the other results.

**Table 3:** Bibliometric characteristics of the selected studies

Authors-Reference	Country - Year of publication	Title	Type	Keywords
Du Q, Ma X, Wang S, Zhou S, Luo C, Tian K, Fei W, Liu X. [5]	China,2022	A digital intervention using virtual reality helmets to reduce dental anxiety of children under local anesthesia and primary teeth extraction: A randomized clinical trial.	RCT	Child behavior, Dental anxiety, Distract systems, Pain perception, Virtual reality exposure therapy
Ran L, Zhao N, Fan L, Zhou P, Zhang C, Yu C [3]	China,2020	Application of virtual reality on non- drug behavioral management of short- term dental procedure in children.	RCT	Virtual reality, Dental anxiety, Pain, Short- term dental procedure
Bagher SM, Felemban OM, Alandijani AA, Tashkandi MM, Bhadila GY, Bagher AM [6]	Saudi Arabia,2023	The effect of virtual reality distraction on anxiety level during dental treatment among anxious pediatric patients: A randomized clinical trial.	RCT	Virtual reality, Dental anxiety, Pain, Short- term dental procedure
Singh R, Gupta N, Gambhir N [7]	India,2022	Comparative evaluation of reduction in pain perception using 5% Topical LA vs. Freezed Cone as a preparatory agent for intraoral injection in children and effect of virtual reality distraction as distraction technique.	RCT	Behavior, Children, Dental anxiety, Interventional study, Pain, Virtual reality eye-glasses
Gs G, George S, Anandaraj S, Sain S, Jose D, Sreenivas A, Pillai G, Mol N. [8]	India,2021	Comparative evaluation of the efficacy of virtual reality distraction, audio distraction and tell- show- do techniques in reducing the anxiety level of pediatric dental patients: An in vivo study.	RCT: in vivo study	Audiovisual distraction, Active distraction, Behavior management, Passive distraction Pediatric dentistry
Nunna M, Dasaraju RK, Kamatham R, Mallineni SK, Nuvvula S. [9]	India,2019	Comparative evaluation of virtual reality distraction and counter- stimulation on dental anxiety and pain perception in children.	RCT: Parallel study	Anxiety, Audiovisual distraction, Behavior management, Pediatric patients, Visual Analogic scale, Wong- Baker Faces pain Rating scale
Tailor B, Bargale S, Dave BH, Deshpande AN, Thomas PS, Jain A. [10]	India,2021	Comparision of virtual reality glasses vs. On- screen distraction technique in reduction of pediatric dental anxiety : An in vivo study.	RCT: in vivo study	Pediatric dental care, Pain perception, Parental acceptance, Patient acceptance, Pharmacological management, Tell- show- do (TSD)
Alshatrat SM, Sabarini JM, Hammouri HM, Al-Bakri IA, Al-Omari WM. [11]	Jordany,2021	Effect of immersive virtual reality on pain in different dental procedures in children: A pilot study.	RCT: Pilot study	Counter- stimulation Dental anxiety Distraction Perception, Virtual reality
Shetty V, Suresh LR, Hegde AM. [12]	India, 2019	Effect of virtual reality distraction on pain and anxiety during dental treatment in 5- to 8-year-old children.	RCT	Audiovisual distraction, Behavior, Behavior management, Children, Dental anxiety, Virtual reality glasses
Felemban OM, Alshamrani RM, Aljeddawi DH, Bagher SM [13]	Saudi Arabia, 2021	Effect of virtual reality distraction on pain and anxiety during infiltration anesthesia in pediatric patients: A randomized clinical trial.	RCT	Distraction, Behavior guidance, Children’s dental anxiety
Koticha P, Katge F, Shetty S, Patil DP. [14]	India, 2019	Effectiveness of virtual reality eyeglasses as a distraction aid to reduce anxiety among 6–10 year- old children undergoing dental extraction procedure.	RCT	Audiovisual eyeglasses, Dental treatment Children Distraction technique Anesthesia injection
Sharma Y, Bhatia HP, Sood S, Sharma N, Singh A. [15]	India, 2021	Effectiveness of virtual reality glasses digital screens and verbal command as a method to distract young patients during administration of local anesthesia.	RCT	Virtual reality, Pain, Pediatric, Local Anesthesia
Niharika P, Reddy NV, Srujana P, Srikanth K, Daneswari V, Geetha KS. [16]	India, 2018	Effects of distraction using virtual reality technology on pain perception and anxiety levels in children duringpulp therapy of primary molars.	RCT: in vivo study	Anxiety, Distraction, Infiltration anesthesia, Pain, Virtual reality
Kumari S, Bahuguna R, Garg N, Yeluri R. [17]	India, 2021	Immersive and non- immersive virtual reality distraction on pain perception to intraoral injections.	RCT: in vivo study	Audiovisual distraction eyewear, Dental anxiety, Pulp therapy
Rao DG, Havale R, Nagaraj M, Karobari NM, Latha AM, Tharay N, Shrutha SP. [18]	India, 2019	Assessment of Efficacy of Virtual reality distraction in reducing pain perception and anxiety in children ages 6–10 years: A behavioral Interventional study	A behavioral interventional study	Behavior, Children, Dental anxiety, Interventional study, pain, Virtual reality eye- glasses

### Risk of bias assessment and data synthesis

Although cohort studies were eligible according to the inclusion criteria, none met the eligibility criteria after full-text screening; therefore, only randomized controlled trials and interventional studies were included in the final analysis.

A total of 14 randomized controlled trials (RCTs) and 1 interventional clinical study were included in this review. The Cochrane Risk of Bias 1 (RoB 1) tool was applied to the RCTs, while the ROBINS-I tool was used for the non-randomized interventional study. These assessments evaluated the internal validity of each study across key domains: random sequence generation, allocation concealment, blinding of participants and personnel, blinding of outcome assessment, incomplete outcome data, selective reporting, and other potential sources of bias. The non-randomized study was included due to its relevance in addressing VR distraction in pediatric dentistry; its findings were interpreted with caution and weighted less in the narrative synthesis given the elevated risk of bias.

**Fig. 2:**
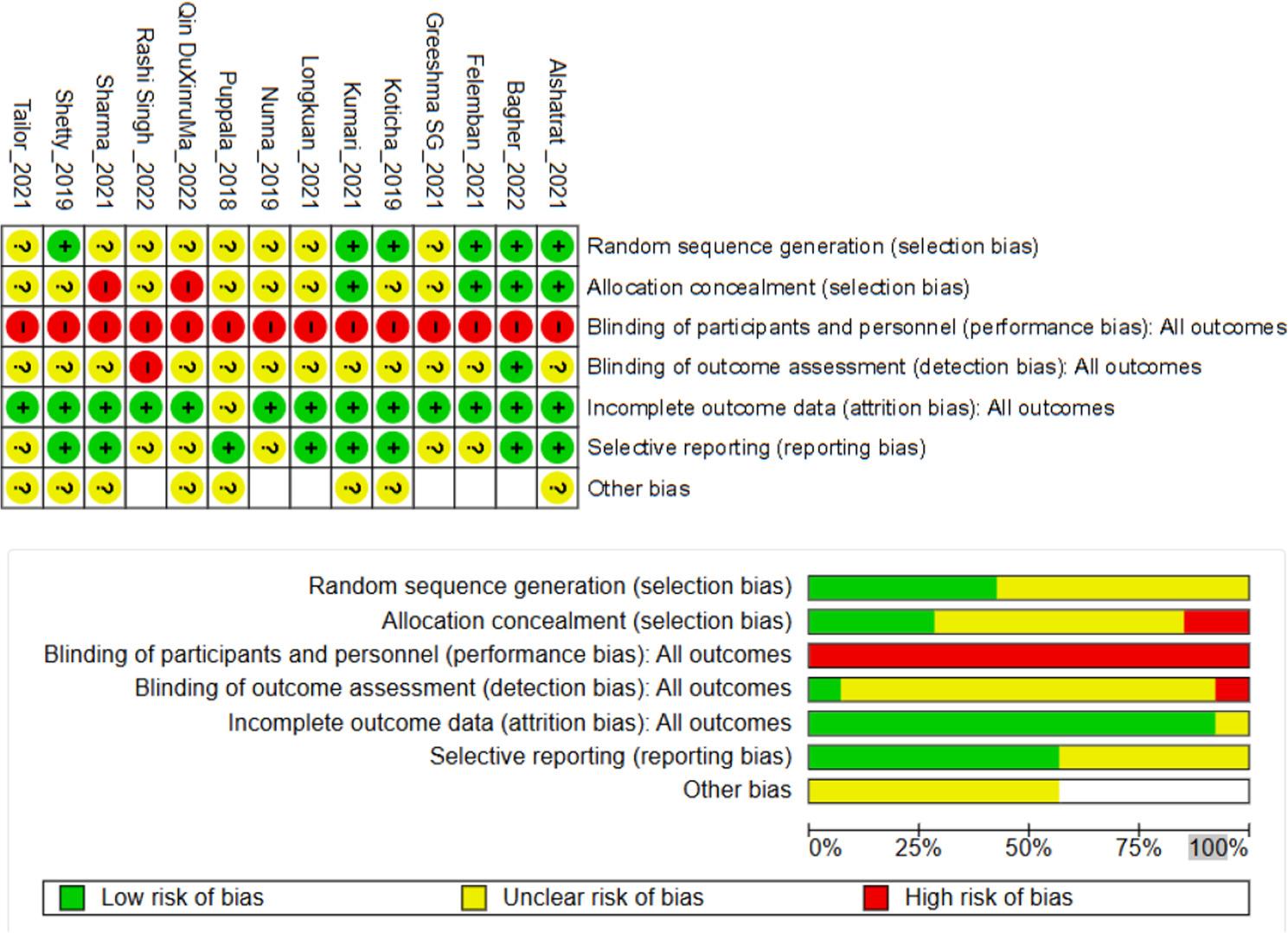
Risk of bias summary across included RCTs. (Cochrane RoB 2 tool)

Two complementary visualizations were prepared to summarize these assessments:

Figure 2 – RoB 1 Bubble Grid: Each bubble represents the risk of bias for a specific domain in a study, with colors indicating low risk (green), some concerns (yellow), or high risk (red). This figure allows a quick visual appraisal of bias patterns across studies.

For the interventional clinical study [19], a separate Table 4 summarize the ROBINS-I assessment, highlighting potential biases related to confounding, selection, intervention classification, outcome measurement, missing data, reporting, and other domains.

**Table 4:** ROBINS-I: Assessment of efficacy of virtual reality distraction in reducing pain perception and anxiety in children ages 6–10 years: A behavioral interventional study [18]

Confounding	Risk Judgment	Justification / Comments
	Serious	The study did not include a control group or adjust for important confounders such as baseline anxiety, previous dental experiences, or individual pain sensitivity.
Selection of participants	Moderate	Only cooperative children (Frankl 3 and 4) were included, limiting representativeness and introducing potential selection bias.
Classification of interventions	Low	The intervention (VR eyeglasses) was clearly defined and consistently applied to all participants.
Deviations from intended interventions	Low	No alternative treatments or co-interventions were reported; all children followed the same procedure.
Missing data	Low	No missing data or dropouts were reported; all measurements were recorded before, during, and after the procedure.
Measurement of outcomes	Moderate	Outcome assessment was not blinded, and subjective measures (Wong-Baker FACES, FLACC) may have introduced bias.
Selection of reported results	Low	All pre-specified outcomes were reported; no evidence of selective reporting.
Overall Risk of Bias	Serious	The lack of a control group and absence of adjustment for major confounders confer a serious overall risk of bias, despite good intervention adherence and complete outcome reporting.

### Summary of bias patterns

Across the 14 RCTs, risk of bias varied across domains as illustrated in Fig. 2.

For random sequence generation, 6/14 studies (43%) were rated as low risk, while 8/14 studies (57%) were rated as having an unclear risk, with no studies judged at high risk.

For allocation concealment, 5/14 studies (36%) were rated as low risk, and 9/14 studies (64%) as having an unclear risk, with no high-risk ratings observed.

Unclear to high risk of bias was most frequently observed in blinding of participants and personnel (performance bias), which is inherent to virtual reality and other distraction-based interventions due to their visible and interactive nature. In this domain, 1/14 studies (7%) was rated as low risk, 4/14 studies (29%) as having unclear risk, and 9/14 studies (64%) as high risk. This limitation is particularly relevant for subjective outcome measures such as pain and anxiety scales.

Similarly, blinding of outcome assessment (detection bias) showed substantial risk, with 1/14 studies (7%) rated as low risk, 4/14 studies (29%) as having unclear risks and 9/14 studies (64%) as high risk. This is particularly relevant for subjective outcome measures such as pain and anxiety scales, where assessor awareness of the intervention may influence scoring.

For incomplete outcome data (attrition bias), most studies demonstrated a low risk of bias, with 12/14 studies (86%) rated as low risk, 2/14 studies (14%) as having unclear risk, and no studies classified as high risk.

For selective reporting, 11/14 studies (79%) were rated as low risk, 2/14 studies (14%) as having unclear risk, and 1/14 study (7%) as high risk.

Finally, other sources of bias were generally unclear in most studies, mainly due to insufficient reporting of design-specific limitations such as crossover procedures, operator effects, or the influence of previous dental experience.

Overall, these results indicate that while attrition and reporting biases were generally low, major concerns remain regarding performance and detection bias, largely due to the inherent impossibility of blinding in VR-based interventions.

Detailed domain-specific risk of bias assessments are provided in supplementary File 2.

Overall, these assessments indicate that while objective outcomes and complete follow-up were generally reliable, the inherent impossibility of blinding in VR-based interventions introduces unavoidable performance and detection bias, particularly affecting subjective outcomes. These limitations should be considered when interpreting the effectiveness of VR and other distraction techniques for pediatric pain and anxiety. Overall, 0% of RCTs were judged to have a low overall risk of bias, while the majority presented unclear risk or high risk across one or more domains.

### Study outcomes

A total of 15 studies, mainly randomized clinical trials and one interventional study, met the eligibility criteria. 1168 children aged 4 to 15 years were included in the review. Publication years ranged from 2019 to 2023.10 of the 15 studies were conducted in India. The main results are presented in (Table 5).

**Table 5:** Study outcomes across included RCTs and interventional study

Ref	Comparison arm (s)		VR intervention (arm)	Study Subject		Type of treatment	Results	
				Age (Years)	Initial level of cooperation			
5	Communication, Tell-Show-do technique, positive reinforcement		VR	4–9	CFSS-DS: Cooperative children – non anxious	Milk Tooth extraction under Local anesthesia	Physiological parameters	Not reported
							Anxiety	↓ significant after treatment in VR group ; No significant change in TSD
							Pain - (WBFS)	**↓** VR < TSD
							Behavior	Not reported
							Adverse effects (Motion)	No significant difference
3	Tell-Show-do technique		VR	4–8	Cooperative children – Non anxious	Caries treatments, extraction of milk tooth, drainage of abscesses	Physiological parameters	Not reported
							Anxiety	↓ significant (VR vs. TSD)
							Pain	↓ (WBFPS ; VR < control)
							Behavior	↑ cooperation
							Adverse effects	Not reported
6	Audiovisual Distraction		VR	6–14	Anxious children, Negative: Frankl III	Prophylactic treatments	Physiological parameters (HR) (SCL)	↑ in VR group vs. AV group (Gr-VR > Gr-AV); SCL ↓ in VR group vs. AV group (Gr-VR < Gr-AV)
							Anxiety	No significant difference (Gr-VR = Gr-AV)
							Behavior	No significant difference (Gr-VR = Gr-AV)
							Pain	Not reported
							Adverse effects	Not reported
7	VR		Without VR distraction	6–11	Children positive or definitively positive – Frankl III-IV	Dental extractions, Temporary treatments on temporary teeth under Local Anesthesia	Physiological parameters	Not reported
							Anxiety	Not reported
							Behavior	Not reported
							Pain	↓ with pre-anesthesia ice cube + VR distraction
							Adverse effects	Not reported
8 99	TSD	Audio distraction	VR	6–8	Children with moderate anxiety: MDAS	Extraction of mandibular teeth under local anesthesia	Physiological parameters (HR; O2 saturation)	HR: ↓ in VR group vs. control; O₂ saturation: ↑ in Audio group (max)
							Anxiety	Not reported
							Behavior	Not reported
							Pain	Not reported
							Adverse effects	Not reported
9	Vibro-tactile counter-stimulation		VR	7–11	Children with anxiety – Frankl Scale	Pulp treatments, extractions of decidual teeth under local anesthesia	Physiological parameters	Not reported
							Anxiety (VBARS)	↓ significant in VR + VACS group vs. ↓ non-significant in Counter-stimulation group
							Behavior	Not reported
							Pain (Wong Baker Face Pain Scale)	↓ less in Counter-stimulation group < VR group
							Adverse effects	Not reported
10	Screen based audiovisual distraction		VR	4–8	Not mentioned	Cavity removing under local anesthesia and pulp treatments or extraction of temporary teeth	Physiological parameters (HR, O2 sat)	HR ↑ in Screen group vs. VR group; O₂ saturation: significant difference
							Anxiety (Venham scale)	↓ clear in VR group
							Behavior	Not reported
							Pain	Not reported
							Adverse effects	Not reported
11	No Distraction		VR	5–12	Not mentioned	Prophylactic treatments (without anesthesia)	Physiological parameters	Not reported
							Anxiety	Not reported
							Behavior (FLACC scale)	Crying & screaming 43% without distraction vs. 6% in VR group
							Pain (AVS)	↓ significant in VR group
							Adverse effects	Not reported
12	Psycho behavioral management techniques		VR	5–8	SCARED Questionnaire < 25: Children predisposed to anxiety	Pulp treatments	Physiological parameters (SCL)	Salivary cortisol level ↓ G2 < G1
							Anxiety (MCDAS)	↓ 18,3% in G1 vs. ↓ 19% in G2
							Behavior	Not reported
							Pain (WBFPRS)	↓ G2 < G1 (2,42 < 5,6)
							Adverse effects	Not reported
13	Screen based audio visual distraction		VR	6–12	Frankl scale: Positive Children	Pulp treatments, Extraction of temporary teeth under local anesthesia	Physiological parameters (HR)	↑ in VR group vs. screen group
							Anxiety	Not reported
							Behavior (FLACC scale)	Max scores: young subjects +females regardless of distraction setup
							Pain	Not reported
							Adverse effects	Not reported
14	No distraction		VR	6–10	Frankl scale: Children Positive or definitively positive	Extractions of two temporary molars	Physiological parameters (HR)	↑ significant in group without distraction
							Anxiety (VBARS)	↑ significant in VR group
							Behavior	Not reported
							Pain	Not reported
							Adverse effects	Not reported
15	G A Classical verbal command approach	Gr B Distraction by virtual reality headset	Gr C Audiovisual distraction via touch screen	4–8	Frankl scale: Children Positive or definitively positive	Extractions or pulp treatment under local anesthesia	Physiological parameters	Not reported
							Anxiety	Not reported
							Behavior	Not reported
							Pain (FLACC scale)	Maximum scores : Gr A > Gr C > Gr B
							Adverse effects	Not reported
16	No VR distraction		VR	4–8	SCARED Questionnaire: Children without anxiety disorders	Pulp treatment in temporary molars	Physiological parameters	Not reported
							Anxiety	Not reported
							Behavior	Not reported
							Pain (FPS)	↓ in VR session vs. without VR session
							Adverse effects	Not reported
17	Gr II Distraction by non immersive VR headset (without controllers)		Gr I Distraction by immersive VR headset (with controllers)	6–12	Positive and definitively positive: Frankl Scale III-IV	Extraction and pulp treatment under local anesthesia	Physiological parameters	Not reported
							Anxiety	Not reported
							Behavior	Not reported
							Pain (VAS), (WBFRS)	↑ Group II (2,72 ± 0,99) vs. Group I (0,75 ± 0,88), ↑ Group I (2,78 ± 1,097) vs. Group II (0,82 ± 1,104)
							Adverse effects	Not reported
18	Distraction by VR headset			6–10	Not mentioned	Restorative Treatments	Physiological parameters (HR)	↓ decrease during treatment, then ↑ increase at end
							Anxiety	Not reported
							Behavior	Not reported
							Pain (FLACC scale), (WBFRS)	WBFPRS: ↓ significant; FLACC scale ↓ decrease in pain perception
							Adverse effects	Not reported

*Abbreviations*: *VR* Virtual Reality, *TSD* Tell-Show-Do, *FLACC* Face, Legs, Activity, Cry, Consolability scale, *MCDAS* Modified Child Dental Anxiety Scale, *WBFPRS* Wong-Baker FACES Pain Rating Scale, *HR* Heart Rate, *SCL* Salivary Cortisol Level, *VAS* Visual Analogic Scale, *VBARS* Venham’s Anxiety and behavioral rating scale, *AV* Audiovisual, *Gr* Group, *AVS* Analogic Visual Scale, *FPS* Face Pain Scale

### Discussion: summary of available data

Anxiety remains a significant obstacle to successful pediatric dental care, as it can interfere with cooperation and treatment outcomes. To address this, several techniques have been proposed to reduce anxiety, aiming to improve the child’s adaptability and acceptance of care, as recommended by the American Academy of Pediatric Dentistry (AAPD). Among non-pharmacological strategies, distraction has emerged as an effective approach, particularly virtual reality (VR), which has gained increasing attention in dentistry. Initially developed for education and entertainment, VR is now used as a psycho-behavioral tool to reduce anxiety in children during dental procedures, with its application expanding across multiple medical fields.

Effectiveness of these strategies is typically measured using validated behavioral and self-reported scales for anxiety, observational assessments of behavior, and standardized tools for pain. This combination provides an integrated view of the child’s response to dental procedures, allowing comparison between VR, traditional psycho-behavioral techniques, and absence of distraction. Such data may inform clinical decision-making, although their direct transposition to routine clinical practice should be interpreted with caution due to the low certainty of evidence.

### Effectiveness of VR distraction compared to other methods

Overall, VR distraction appears to be a promising tool that may help reduce anxiety and pain perception in children, although the certainty of evidence remains low [13, 14]; however, these findings should be interpreted cautiously due to moderate risk of bias, mainly related to lack of blinding and small sample sizes. Additional contextual evidence also supports the potential usefulness of VR, although it does not allow firm conclusions to be drawn [19, 20].

Compared with passive audiovisual distraction, VR was reported to show greater effectiveness in some included studies [10], while contextual literature has reported similar trends (22,23). However, these findings were derived from studies with moderate risk of bias, and other included studies reported no significant differences [6], limiting the certainty of comparative effectiveness.

VR was associated with possible reductions in some physiological parameters, including salivary cortisol in selected studies, although findings remain inconsistent [6] and improved oxygen saturation, as also reported in contextual literature (21), although some studies reported no significant improvement in pain perception, as assessed by the FLACC scale [13] or Venham scale.

Both immersive and non-immersive VR systems have been reported to provide beneficial effects in pain management, although findings remain heterogeneous [16]. In comparisons with vibro-tactile counter-stimulation, VR was reported to be more effective for anxiety reduction, whereas vibro-tactile stimulation sometimes provided superior pain relief [9]. Combining VR with other distraction techniques may represent a potential strategy, although this is based on limited and heterogeneous evidence [9, 13, 16].

These conclusions are tempered by risk-of-bias assessments across the included studies (Fig. 2) [6, 9, 10, 13, 14, 16], with studies at moderate or serious risk contributing less weight to the overall interpretation. Overall, the certainty of evidence for VR distraction was rated as low according to GRADE (Table 6), which limits the ability to draw strong clinical conclusions or infer superiority between techniques.

Taken together, the available evidence remains limited, heterogeneous, and methodologically constrained across the included studies [6, 9, 10, 13, 14, 16], and therefore no definitive conclusions regarding the superiority of VR over other distraction techniques can currently be drawn due to the low certainty and heterogeneity of the evidence.

### Analysis of extracted data

Most studies originated from India (10/15 studies; Table 3) [7–10, 12, 14–18], although research teams varied across articles. This geographic concentration may partly reflect regional research priorities, accessibility of VR technology, or publication patterns. However, the predominance of studies conducted within a single country may limit the external validity of the findings. Cultural norms regarding child behavior, parental expectations, pain expression, and behavioral management strategies in dental settings may differ across regions. Consequently, children’s responses to distraction techniques such as VR may not be directly generalizable to populations with different sociocultural backgrounds or healthcare systems.

The children included ranged in age from 4 to 15 years (Table 5), reflecting a broad spectrum of developmental stages and behavioral responses. Over 60% of studies focused on children older than six years (Table 5), as younger children (4–6 years) often experience pain more intensely and may have less ability to cooperate with distraction techniques [6–9, 12, 13, 16, 17]. Cultural differences in child autonomy, communication, and behavioral expectations may also influence age-related responses to VR across settings.

### VR distraction may also benefit children and early adolescents (up to 15 years old), although this subgroup was underrepresented in the included studies

All included children were generally healthy (Table 5). Children with severe behavioral or communication disorders (e.g., ASD), as well as those unable to use the VR device due to speech, hearing, or vision difficulties, were commonly excluded where specified by study eligibility criteria. Other absolute contraindications included epilepsy or vestibular disorders. This selective inclusion of generally healthy and cooperative children further restricts the applicability of findings to more diverse pediatric populations, including those with special healthcare needs.

The child’s initial level of cooperation is an important predictor of behavior during care. Most studies included children classified as having positive or definitively positive behavior (III–IV) where behavior scales were reported (Table 5), raising the question of whether VR distraction is necessary in children who are already predisposed to cooperate, although anxiety reduction was still observed in several studies. However, several studies still demonstrated significant reductions in anxiety (e.g., ↓ CFSS-DS, ↓ MCDAS) and pain scores (e.g., ↓ WBFPS, ↓ FLACC, ↓ VAS) in VR groups compared with control or conventional behavioral techniques.

Variation in assessment scales, number of treatment sessions, and types of dental procedures (from non-invasive prophylaxis to extractions) further complicates direct comparisons but highlights the overall trend that VR distraction may provide additional benefit even in cooperative children across various dental procedures, although the magnitude of effect remains uncertain. For example, significant reductions in pain were reported in multiple studies (e.g., WBFPRS significantly lower in VR vs. control; FLACC scores reduced from 43% crying/screaming without distraction to 6% with VR [11]), and anxiety reductions were also observed in several trials (e.g., significant decrease in Venham scale and CFSS-DS in VR groups compared with TSD techniques [5, 3], while others reported significant reductions in anxiety and pain compared with audiovisual distraction) [10] .However, these findings should be interpreted with caution given the heterogeneity among the included studies [6, 9, 10, 13, 14, 16].

Taken together, the geographic concentration of studies and the selective inclusion criteria suggest that caution is warranted when extrapolating these findings to broader international or more behaviorally diverse pediatric populations.

### Heterogeneity and data synthesis and quality of evidence

The included studies exhibited substantial clinical, methodological, and outcome heterogeneity, which precluded a quantitative meta-analysis. Anxiety and pain were measured using a variety of validated instruments (MCDAS[f], CFSS-DS, VPT, VBARS, FLACC, WBFPS, VAS), differing in scoring systems, timing, and interpretation.

Intervention modalities varied widely, including immersive and non-immersive VR [17], audiovisual screens [6, 10, 13, 15], vibro-tactile distraction [9], and verbal or behavioral techniques [3, 5, 7, 12, 15], with differences in session number and duration. Participants ranged from 4 to 15 years, with varying baseline anxiety and behavioral status.

Importantly, several studies demonstrated superior outcomes in VR groups, including significant reductions in pain scores, with lower WBFPRS values observed in VR compared with tell-show-do and verbal techniques, as well as reductions in FLACC scores across multiple trials [11, 15, 18]. Reductions in anxiety levels were also reported, with lower CFSS-DS, MCDAS, and Venham scores in VR groups compared with control or conventional behavioral management approaches [5, 3, 12, 10]. In addition, improvements in cooperation were observed in some studies, with higher cooperative behavior scores reported in VR groups compared with control conditions [3, 11]. Finally, physiological benefits were also noted in several trials, including lower heart rate and reduced stress-related markers in patients exposed to VR interventions [6, 12, 18].

The type of dental care also differed, from non-invasive prophylactic treatments [6, 11, 18] to restorative procedures and extractions [3, 5, 7–10], further contributing to clinical heterogeneity. These variations in procedure type likely influenced baseline pain and anxiety levels, contributing to variability in observed effects.

However, not all studies demonstrated consistent superiority of VR, with some reporting no significant difference between VR and audiovisual distraction [6] or mixed physiological responses (e.g., HR variations depending on comparator). Therefore, while trends generally favor VR, the magnitude and consistency of effect remain uncertain.

**Table 6:** GRADE assessment of the quality of evidence for VR distraction in pediatric dentistry

Outcome	Risk of bias	Inconsistency	Indirectness	Imprecision	Overall certainty (GRADE)
Anxiety reduction	Serious	Serious	Not serious	Serious	Low
Pain perception reduction	Serious	Serious	Not serious	Serious	Low
Behavioral improvement/compliance	Serious	Serious	Serious	Serious	Low

Methodological quality varied across studies, with risks of performance, detection, and selection bias due to lack of blinding, unclear allocation concealment, and small sample sizes in some trials, as summarized in the RoB 2 and ROBINS-I assessments (Fig. 2). The high risk of performance and detection bias is largely inherent to VR interventions, which may lead to an overestimation of effects, particularly for subjective outcomes such as pain and behavior scores. This limitation is particularly important for subjective outcome measures (e.g., WBFPS, FLACC), where knowledge of the intervention may systematically influence reported or observed outcomes, potentially leading to an overestimation of benefits.

Studies with moderate or high risk of bias were therefore interpreted with caution and given reduced weight in the narrative synthesis. The non-randomized interventional study, rated as serious risk of bias due to confounding and absence of a control group, provides limited inferential value and was considered accordingly in the interpretation of findings [18].

These methodological limitations, together with heterogeneity in outcome measures and intervention protocols, reduced confidence in individual study results and precluded meta-analysis; consequently, a narrative synthesis was conducted.

The reliance on subjective outcome measures and the visible nature of VR interventions introduce performance and detection bias, which should be considered when interpreting the results. In addition, the predominance of non-randomized and heterogeneous trial designs limits causal inference regarding the effectiveness of VR distraction in pediatric dentistry.

Overall, the certainty of evidence remains low (GRADE), and no firm conclusions regarding the clinical superiority of VR interventions can be drawn at present. Future high-quality randomized controlled trials with standardized outcome measures and rigorous methodological design are needed.

### Practical considerations for VR use in pediatric dentistry

Virtual reality (VR) can be a useful tool in pediatric dentistry, but its use should be adapted to each child. t appears most suitable for children aged 6 and older, based on the age distribution of included studies rather than direct comparative evidence. Before using a VR headset, practitioners are advised to assess the child’s health. Children with serious conditions such as uncontrolled seizures, severe heart or vestibular problems, or uncorrected visual impairments are generally not suitable candidates. Although some studies included participants up to 15 years of age, most samples primarily consisted of younger children. Therefore, conclusions regarding adolescents should be interpreted cautiously, as this subgroup was not specifically analyzed in the included studies. The number and duration of VR sessions varied across the included studies and should be tailored to the child’s tolerance and the type of procedure. VR distraction has been applied across a wide range of treatments, although evidence supporting its effectiveness varies depending on study quality. It is recommended to use standalone headsets appropriate for children, ensure comfort and safety, provide age-appropriate content, and train staff in proper device use. Continuous supervision and thorough cleaning of the headset after each session are essential. Parental consent should always be obtained, and feedback from both the child and their parents should be collected after each session to guide future use.

This systematic review suggests that virtual reality (VR) distraction may represent a non-invasive technique with a generally favorable safety profile reported in included studies, although adverse events were inconsistently reported. VR was reported in several studies to achieve greater reductions in anxiety compared with certain conventional psycho-behavioral methods; however, these findings remain inconsistent and are based on evidence of low certainty.Importantly, these conclusions are tempered by the risk-of-bias assessments, with studies at moderate or serious risk contributing less weight to the overall interpretation. Classic psycho-behavioral approaches remain important for teaching long-term coping skills. The choice of technique should consider the child’s preferences, clinical context, and available resources.

Therefore, strong clinical recommendations or judgments of superiority cannot currently be made. Overall, while VR appears promising, the interpretation of its effectiveness must account for methodological limitations, risk of bias, and study design, highlighting the need for further high-quality trials.

### Limitations

Certain factors may limit the generalizability and interpretation of the review findings. Only studies published in English were included due to the authors’ language proficiency, which may have excluded relevant studies in Portuguese, Spanish, or other languages. Most included studies originated from India, which may limit the applicability of findings to other geographic regions. Additionally, methodological heterogeneity across studies, including differences in anxiety and pain measurement scales, VR interventions, and participant characteristics, combined with risk of bias in individual studies, reduces the overall certainty of the evidence.

Furthermore, the use of different anxiety and pain assessment tools, with varying psychometric properties (e.g., validity and reliability), may affect the comparability of results across studies and contribute to variability in reported outcomes.

No assessment of inter-reviewer agreement (e.g., kappa statistic) was performed, which may limit the reproducibility of the study selection and data extraction processes. In addition, grey literature and trial registries were not searched, which may increase the risk of publication bias, and reference lists of included studies were not systematically screened, which may have led to the omission of relevant studies.

Behavioral outcomes are inherently subjective and therefore susceptible to observer bias, particularly in studies relying on visual or self-reported scales. Moreover, bias is largely unavoidable in behavioral interventions such as virtual reality due to the impossibility of blinding participants and personnel to the intervention, which may further influence outcome assessment.

Although a formal assessment of publication bias was not conducted due to the small number of included studies, the possibility of small-study effects cannot be excluded. Smaller, exploratory studies may be more likely to report positive findings, and selective outcome reporting could contribute to an overestimation of VR distraction’s effectiveness in pediatric dentistry. Additionally, the possibility of publication bias cannot be excluded, as studies reporting positive or significant effects of virtual reality may be more likely to be published than those with negative or inconclusive findings. This may have led to an overestimation of the reported benefits. Grey literature and trial registries were not searched, which may introduce a risk of publication bias.

## Supplementary Information

Supplementary Material 1.

Supplementary Material 2.

## Data Availability

All data generated or analyzed during this systematic review are included in this published article. The datasets analyzed were obtained from publicly accessible databases (PubMed, Cochrane Library, Scopus, and ScienceDirect).

## Abbreviations

AV eyeglasses: Audiovisual eyeglasses
CFSS-DS: Child fear survey schedule dental subscale
FBRS: Frankl Behavior Rating scale
FLACC: Face, Legs, Activity, Cry, Consolability behavioral pain assessment scale
HR: Heart Rate
IANB: Infra alveolar Nerve block
MCDAS: Modified child dental Anxiety scale
Mesh: Mesh Medical Subject Headings
PJS: Pictoral scale
SCARED: Screen for Child Anxiety Related Disorders
SCL: Salivary Cortisol Level
SEM scale: Sound Eye and Motors scale
VAS: Visual Analogic Scale
VBARS: Venham’s Anxiety and behavioral rating scale
VCARS: Venham’s clinical Anxiety rating scale
VPT: Venham picture test
VR: Virtual reality
WBFPS: Wong Baker faces pain scale
