# Supplementary material for: Effect of virtual reality distraction on children’s behavior during dental treatment: a systematic review of the literature
Source: BMC Oral Health. 2026 May 26;26:1390. doi: 10.1186/s12903-026-08646-3 (PMC13455445; doi:10.1186/s12903-026-08646-3)
Supplement: Supplementary file 1 [file 12903_2026_8646_MOESM1_ESM.docx]

**Manuscript ID :** b06f321e-3c98-47ce-8b9b-86e146d1ec04

**Title :** Effect of virtual reality distraction on child behavior during dental treatment

Supplementary file 2

|  | | **QinDuXinruMa 2021** | **Longkuan Ran 2021** | **Bagher 2022** | **Singh 2022** | **Greeshma 2021** | **Nunna 2019** | **Tailor 2021** | **Alshatrat 2021** | **Shetty 2019** |
| --- | --- | --- | --- | --- | --- | --- | --- | --- | --- | --- |
| **Random sequence generation-** Selection bias | Judgment | Unclear risk | Unclear risk | Low risk | Unclear risk | Unclear risk | Unclear risk | Unclear risk | Low risk | Low risk |
|  | Support for judgment | Randomization by coin flip ; details unclear. | Randomization via software ; details insufficient. | Stratified block randomization; method appropriate and clearly described | Envelope randomization; sequence generation unclear. |  | Random numbers used; sequence generation  unclear | Chit method used; sequence generation unclear. | Computer- generated random sequence; reliable  method | Proper randomizatio n; allocation likely  unbiased |
| **Allocation concealment -** Selection bias | Judgment | High risk | Unclear risk | Low risk | Unclear risk | Unclear risk | Unclear risk | Unclear risk | Low risk | Unclear risk |
|  | Support for judgment | No allocation concealment; potential selection bias. | Allocation concealment unclear; potential  selection bias. | Allocation concealed by independent  assistant; prevents foreknowledge | Envelope method; allocation concealment  unclear. | Allocation concealment not reported; potential  selection bias | Allocation concealment not described;  selection bias possible | Allocation by co- investigator; concealment not reported, selection bias  possible. | Sealed, opaque envelopes; allocation concealed. | Allocation concealment not described; predictability unclear. |
| **Blinding of participants and personnel**  **-**  Performance bias | Judgment | High risk | High risk | High risk | High risk | High risk | High risk | High risk | High risk | High risk |
|  | Support for judgment | No blinding; high risk of performance bias. | No blinding of participants/personnel ; subjective outcomes may be influenced, high performance bias risk. | No blinding; may affect subjective outcomes, objective  outcomes less likely biased. | Envelopes and blinding details not reported; allocation  concealment unclear. | No blinding of participants/ personnel ; subjective  outcomes may be influenced. | No blinding ; subjective outcomes  likely influenced. | No blinding; subjective outcomes may be influenced | No blinding; participants and clinician  aware of intervention | No blinding; VR  intervention known, high  performance bias risk |
| **Blinding of outcome assessment –** Detection bias | Judgment | Unclear risk | Unclear risk | Low risk | High risk | Unclear risk | Unclear risk | Unclear risk | Unclear risk | Unclear risk |
|  | Support for judgment | Assessor blinding not reported; risk of detection bias unclear. | Assessor blinding not reported; validated  scales used, detection bias unclear. | Evaluators rated videos independently; blinding not  stated, objective outcomes unaffected | Assessor blinding not reported; subjective outcomes, detection bias likely. | Objective outcomes less biased; facial scale assessor blinding not reported, detection bias possible | Assessor blinding not reported for subjective  scales; objective outcomes less biased | PI/statistician blinded; subjective  scales  assessed by unblinded co- investigator,  detection bias possible | External observer for FLACC; self- reported  scales may introduce detection bias | Self-reported anxiety/pain; objective cortisol less biased, detection bias possible. |
|  | Judgment | Low risk | Low risk | Low risk | Low risk | Low risk | Low risk | Low risk | Low risk | Low risk |

| **Incomplete outcome data –** Attrition bias | Support for judgment | Low and balanced missing data; unlikely  to bias results. | Low dropout; reasons reported; unlikely to bias results. | No post- randomization dropouts; minimal attrition bias. | No dropouts; all participants analyzed, low attrition bias | No dropouts; all participants analyzed, low attrition bias | No dropouts; all participants  analyzed, low attrition bias | No dropouts; all participants  analyzed, low attrition bias | No loss to follow-up; all data analyzed,  low attrition bias | No dropouts; all participants  analyzed, low attrition bias |
| --- | --- | --- | --- | --- | --- | --- | --- | --- | --- | --- |
| **Selective reporting –** Reporting bias | Judgment | Unclear risk | Low risk | Low risk | Unclear risk | Unclear risk | Unclear risk | Unclear risk | Low risk | Low risk |
|  | Support for judgment | No protocol reported; selective reporting risk unclear. | All pre-specified outcomes reported; low risk of selective reporting. | All pre-specified outcomes reported; low  selective reporting risk. | No protocol reported; selective  reporting risk unclear. | Outcomes reported; no protocol,  selective  reporting risk unclear. | No protocol reported; selective  reporting risk unclear | No protocol reported; selective  reporting risk unclear | All planned outcomes reported; low selective reporting risk. | All pre- specified outcomes reported; low selective  reporting risk |
| **Other bias** | Judgment | NA | NA | NA | NA | N/A | N/A | Unclear risk | Unclear risk | Unclear risk |
|  | Support for judgment |  |  |  |  |  |  | Small sample, short follow- up, repeated measures may bias results. | Minor age imbalances and prior dental experience may slightly bias results. | Single- operator and prior dental experience may bias results; gender  distribution balanced. |

|  | | **Felemban 2021** | **Koticha 2019** | **Sharma 2021** | **Puppala 2018** | **Kumari 2021** |
| --- | --- | --- | --- | --- | --- | --- |
| **Random sequence generation-** Selection bias | Judgment | Low risk | Low risk | Unclear risk | Unclear risk | Low risk |
|  | Support for judgment | Computer-generated randomization; allocation likely unbiased | Split-mouth random assignment; sequence generation likely unbiased | Chit system used; sequence generation details unclear. | Stratified randomization reported; sequence generation details unclear. | Random allocation by colored balls; sequence generation unpredictable  and random |
| **Allocation concealment**  **-**  Selection bias | Judgment | Low risk | Unclear risk | High risk | Unclear risk | Low risk |
|  | Support for judgment | Allocation concealed by independent dental assistant until enrollment | Randomization details and concealment unclear; potential selection bias | Allocation concealment not described; high risk of selection bias | Allocation concealment not described; potential foreknowledge of  assignment | Opaque bag used; allocation concealed, risk of selection bias low |
| **Blinding of participants and personnel -** Performance bias | Judgment | High risk | High risk | High risk | High risk | High risk |
|  | Support for judgment | No blinding; VR visibility may influence behavior and perceived pain. | No blinding; VR visibility may affect behavior and physiological responses | No blinding; participants and clinicians aware, may influence behavior and pain. | No blinding; awareness of VR may influence behavior, anxiety, and pain | No blinding; participants and operators aware, may influence pain and anxiety |
|  | Judgment | Unclear risk | Unclear risk | Unclear risk | Unclear risk | Unclear risk |

| **Blinding of outcome assessment –** Detection bias | Support for judgment | Outcome assessors not blinded; subjective pain scales used, objective HR less biased | Self-reported anxiety; assessor blinding unclear, objective  measures less biased | No blinding; awareness of intervention may affect behavior and pain | Self-reported pain/anxiety; assessor blinding not reported, detection bias possible | Self-reported outcomes; assessor blinding not reported, detection bias possible. |
| --- | --- | --- | --- | --- | --- | --- |
| **Incomplete outcome data –**  Attrition bias | Judgment | Low risk | Low risk | Low risk | Unclear risk | Low risk |
|  | Support for judgment | No dropouts reported; all randomized participants analyzed | No dropouts; all participants analyzed, low attrition bia | FLACC scale used; assessor not blinded, possible detection bias | Balanced dropouts; no ITT analysis reported, some attrition bias concern. | No dropouts; all participants analyzed, low attrition bias |
| **Selective reporting –**  Reporting bias | Judgment | Unclear risk | Low risk | Low risk | Low risk | Low risk |
|  | Support for judgment | No protocol/registration; selective reporting risk unclear. | All pre-specified outcomes reported; low selective reporting risk | All outcomes reported as planned; low selective  reporting risk. | All prespecified outcomes reported; low selective reporting risk | All prespecified outcomes reported; low selective reporting risk |
| **Other bias** | Judgment | NA | Unclear risk | Unclear risk | Unclear risk | Unclear risk |
|  | Support for judgment |  | All pre-specified outcomes (VPT, pulse, oxygen saturation) were fully reported. | Cross-sectional design, age differences, operator/assessor variability, and prior visits may bias results. | Crossover design; possible carryover effects, behavioral conditioning, single- operator study may bias  results. | Subjective outcomes, multiple procedures, short-term assessment may bias results |
