# Supplementary material for: Effect of virtual reality distraction on children’s behavior during dental treatment: a systematic review of the literature
Source: BMC Oral Health. 2026 May 26;26:1390. doi: 10.1186/s12903-026-08646-3 (PMC13455445; doi:10.1186/s12903-026-08646-3)
Supplement: Supplementary file 2 [file 12903_2026_8646_MOESM2_ESM.docx]

Manuscript ID : b06f321e-3c98-47ce-8b9b-86e146d1ec04

Title : Effect of virtual reality distraction on child behavior during dental treatment

- **Supplementary material 1 -**

Pubmed

| Database | Date of search | Search strategy | Filters | Results |
| --- | --- | --- | --- | --- |
| PubMed | July 2023 | (“Virtual Reality”[Mesh] OR “virtual reality”) AND (“Dental Anxiety”[Mesh] OR dental anxiety OR dental fear OR dental phobia) AND (child* OR pediatric OR paediatric) | English, Clinical Trial, Randomized Controlled Trial, Comparative Study, Humans | 13 |

Cochrane Library

| Database | Date of search | Search strategy | Filters | Results |
| --- | --- | --- | --- | --- |
| Cochrane Library | July 2023 | (“Virtual Reality” OR virtual reality) AND (“Dental Anxiety” OR dental anxiety OR dental fear) AND (child* OR pediatric OR paediatric) | English, Clinical Trials | 6 |

Science Direct

| Database | Date of search | Search strategy | Filters | Results |
| --- | --- | --- | --- | --- |
| ScienceDirect | July 2023 | (“virtual reality”) AND (“dental anxiety” OR “dental fear”) AND (child* OR pediatric dentistry OR dental care for children) | Medicine and Dentistry, English, Research articles, Clinical studies | 40 |

Scopus

| Database | Date of search | Search strategy | Filters | Results |
| --- | --- | --- | --- | --- |
| Scopus | July 2023 | TITLE-ABS-KEY (“virtual reality”) AND TITLE-ABS-KEY (“dental anxiety” OR “dental fear”) AND TITLE-ABS-KEY (child* OR pediatric OR paediatric) | Dentistry subject area, English, Clinical studies | 135 |

Google scholar

| Database | Date of search | Search strategy | Filters | Results |
| --- | --- | --- | --- | --- |
| Google Scholar | July 2023 | “virtual reality” AND “dental anxiety” AND (child OR pediatric OR paediatric) | English, Clinical studies | 42 |
